# Comparative Genomic Analysis of *Brevibacillus brevis*: Insights into Pan-Genome Diversity and Biocontrol Potential

**DOI:** 10.3390/microorganisms13112456

**Published:** 2025-10-27

**Authors:** Wenbo Yang, Qiang Bao, Yuanjiang Wang, Lei Xiao, Zexuan Zeng, Lingyun Zhou, Hui Yang

**Affiliations:** 1 Hunan Tea Research Institute, Hunan Academy of Agricultural Sciences, Changsha 410125, China; hncysywb@hunaas.cn (W.Y.); hncysbq0703@hunaas.cn (Q.B.); hncyswyj@hunaas.cn (Y.W.); hncysxl@hunaas.cn (L.X.); zengzexuan@hotmail.com (Z.Z.); 2 Yuelushan Laboratory, Changsha 410128, China

**Keywords:** *Brevibacillus brevis*, comparative genomics, pan-genome analysis, secondary metabolites, biosynthetic gene clusters

## Abstract

The promising biocontrol agent *Brevibacillus brevis* is a broadly dispersed bacterium exhibiting significant antibacterial properties against plant diseases. This study conducted a comprehensive comparative genomic analysis of 25 *B. brevis* strains to examine their taxonomic classification, genetic diversity, and biocontrol potential. The genome sizes, excluding strain NEB573, varied from 5.95 to 6.73 Mb, with GC content between 47.0% and 47.5%. Notably, strain NEB573 exhibited distinct genomic characteristics based on Average Nucleotide Identity (ANI), digital DNA-DNA hybridisation (dDDH), and phylogenetic analyses, suggesting it may represent a novel *Brevibacillus* species pending additional phenotypic confirmation. The remaining 24 strains were grouped into six phylogenetic clades. The pan-genome study demonstrated significant genomic flexibility, demonstrating an open architecture with 2855 core gene families (33.08%) and 1699 distinct genes. Functional annotations indicated that unique genes were enriched in tasks related to DNA repair and environmental adaptation, while core genes predominantly participated in amino acid metabolism and transcription. The examination of biosynthetic gene clusters (BGCs) identified multiple antimicrobial compounds, such as gramicidin and tyrocidine, which have been reported to exhibit both antibacterial and antifungal activities, thereby underscoring the broad-spectrum biocontrol potential of *B. brevis*. These findings endorse the application of biocontrol in sustainable plant disease management and offer novel perspectives on its genetic basis in *B. brevis*. Future investigations of its metabolic repertoire may unveil novel agro-biotechnological applications.

## 1. Introduction

The genus *Brevibacillus* comprises Gram-positive or Gram-variable bacteria with remarkable environmental adaptability, enabling colonization of diverse ecological niches such as soil, marine environments, and plant and animal hosts [1]. Initially classified within the genus *Bacillus* because of shared morphological and physiological traits, these species were later reassigned to *Brevibacillus* based on 16S rRNA gene sequences and phylogenetic analyses [2,3]. This taxonomic revision refined our understanding of evolutionary relationships among related genera. As of 10 October 2025, the List of Prokaryotic names with Standing in Nomenclature (https://lpsn.dsmz.de/genus/brevibacillus) records 34 validly published species in the genus *Brevibacillus* [4].

*Brevibacillus brevis*, the type species of the genus, is well known for producing the peptide antibiotics gramicidin A and gramicidin S [5]. Because of its robust sporulation, metabolic versatility, and strong antimicrobial activity, *B. brevis* has attracted attention as a promising biocontrol agent for sustainable agriculture, offering an environmentally friendly alternative to chemical pesticides [3,6]. Its biocontrol efficacy derives mainly from the biosynthesis of diverse antimicrobial metabolites—including enzymes such as cellulase and chitosanase [7], and non-ribosomal peptides such as surfactin [8], tyrocidine [9], edeine [10], and gramicidin [11]—which disrupt microbial membranes and inhibit pathogen growth. These metabolites contribute to ecological competitiveness and suppression of plant diseases. Nevertheless, most previous studies have focused on individual strains, providing limited insight into the genetic diversity and comparative genomics underlying these traits (Table 1).

In a recent study, Du et al. (2024) performed a pan-genome analysis of nine high-quality complete genomes of *B. brevis*, offering fundamental insights into species-level genomic diversity [12]. However, the limited sample size (representing only 36% of known sequenced *B. brevis* genomes as of 20 March 2025) and exclusive focus on intraspecific comparisons precluded the integration of broader phylogenetic context within the *Brevibacillus* genus. Thus, a more comprehensive genomic framework is still needed to delineate the boundaries and evolutionary context of *B. brevis*. Comparative genomics has proven powerful for revealing metabolic potential in many bacterial lineages such as *Burkholderia*, myxobacteria, and *Lactobacillus* [13,14,15]. By identifying conserved and strain-specific genes, predicting biosynthetic gene clusters (BGCs), and inferring phylogenomic relationships, such analyses provide a descriptive foundation for understanding functional diversity [6,16,17].

To overcome these limitations and expand upon Du et al. (2024)’s foundational work [12], we performed a systematic comparative genomic analysis of 25 *B. brevis* genomes, employing Average Nucleotide Identity (ANI), digital DNA-DNA hybridisation (dDDH), and phylogenomic reconstruction. Our aims were to (i) clarify taxonomic boundaries and inter-strain relationships, (ii) define the core and accessory genome components, and (iii) catalogue and compare BGCs associated with potential biocontrol functions. We also examined the placement of *B. brevis* within the *Brevibacillus* genus to update its evolutionary context. The resulting dataset and analyses provide an expanded and reproducible genomic resource for *B. brevis*. This framework extends previous pan-genomic work by incorporating a larger number of strains and integrating genus-level phylogenetic information, thereby offering a clearer view of genomic diversity, biosynthetic capacity, and the evolutionary relationships that underpin the ecological and biocontrol potential of *B. brevis*.

## 2. Materials and Methods

### 2.1. Genomic Data Acquisition

To investigate the genomic diversity and biocontrol potential of *B. brevis*, twenty-five *B. brevis* genomes and twenty-seven related *Brevibacillus* species were acquired from the NCBI Genome Database (accessed 20 March 2025). For phylogenetic analysis, genomes were selected prioritizing type strains where available; for species lacking type strain genomes, high-quality non-type strain genomes were included (Supplementary Table S1). CheckM v1.2.3 [18] was used to assess genome completeness and contamination based on lineage-specific marker genes. The inclusion of related genomes facilitated comparative assessment of taxonomic and functional characteristics of *B. brevis*. Subsequent analysis focused on twenty-five *B. brevis* genomes filtered for gene completeness ≥90% and contamination ≤5%, ensuring robust genetic data for comparative research.

### 2.2. Phylogenetic Analysis

Phylogenomic placement and taxonomic assignment of all genomes were performed using GTDB-Tk v2.4.1 [19] with the GTDB RS226 reference database. This step assigned genomes to species within the *Brevibacillus* genus based on standardized genomic metrics. The concatenated alignment generated by GTDB-Tk was used to construct a Maximum Likelihood phylogeny with RAxML-NG v1.2.2 [20], employing the best-fit substitution model selected by ModelFinder. Branch support was evaluated via 1000 bootstrap replicates (*Bacillus subtilis* 168; Supplementary Table S1 served as the outgroup). The resulting tree was visualized and annotated using iTOL v7.2.1 [21]. Detailed operational commands and the Newick-format tree file are provided in the Supplementary Methods S1 and the Supplementary File S1.

Strains were classified into lineages following Johnson & Dunlap (2019) [22], which defines: established species (type strains, marked “T” in figures/tables), genomospecies (non-type strains meeting genomic thresholds but lacking formal species designation). This hierarchical framework was applied to all analyzed strains.

### 2.3. Pan-Genome and Comparative Genetic Diversity of Brevibacillus Brevis Based on ANI and DDH Analysis

Average Nucleotide Identity (ANI) values were calculated using FastANI v1.33 [23] with default parameters (Supplementary Methods S1), performing all-vs-all pairwise comparisons to generate a similarity matrix to delineate taxonomic boundaries among *B. brevis* strains, followed by digital DNA-DNA hybridisation (dDDH) values computed via the online GGDC tool v3.0 [24] with default parameters. The resulting ANI and dDDH matrices were imported into TBTools v1.098 [25], where heatmaps were generated using the Heatmap module, color gradients, and scaled ranges (90–100% for ANI, 50–100% for dDDH) to emphasize genomic similarities and differences between strains. This approach facilitated a standardized comparison of genetic diversity and taxonomic consistency across the *Brevibacillus* genomes.

### 2.4. Pan-Genome Analysis

Whole-genome annotation was performed using Prokka v1.14.6 [25] was employed with default parameters (Supplementary Methods S1) to predict coding sequences and generate protein FASTA files. The Bacterial Pan-Genome Analysis pipeline (BPGA) v1.3 [26] was employed following its official user manual. Gene clustering was performed using USEARCH v11.0.667 with a 50% sequence identity cutoff [19]. Input files were faa annotations from Prokka, and outputs included presence/absence matrices and regression plots. The core genome, comprising genes common to all strains; the accessory genome, consisting of genes found in some but not all strains; and unique genes, which are particular to individual strains.

Following pangenome classification into subsets, in-house scripts were employed to estimate the fitting parameters of Heap’s Law using least squares regression (applied to core genome subsets and singleton genes). Heap’s Law was modeled as y = ax^b^, where y denotes the number of genes of the genome, x denotes the number of analyzed genomes, and b are fitting parameters. The exponent b was used to calculate α = 1−b: When α > 1 (b < 0), the pangenome is classified as closed, indicating no significant increase in novel genes with additional sequencing; when α < 1 (0 < b < 1), the pangenome is open, demonstrating continuous gene acquisition as more genomes are sequenced [27].

### 2.5. Functional Annotation of the Pan-Genome

To elucidate metabolic and ecological functions, functional annotations of core, accessory, and unique genes were performed using EggNOG-mapper v2 [28] with an *E-value* threshold of 0.001, employing orthology-based normalisation from the EggNOG 5.0 database. KEGG Mapper/BLASTKOALA [29] (https://www.kegg.jp/blastkoala/, accessed 25 March 2025) was employed for KEGG pathway analysis, categorising genes into functional classifications based on KEGG Orthology (KO) terms. This dual annotation technique offered extensive knowledge about the metabolic capability represented by the entire genome of *B. brevis*, particularly for genes associated with biological regulation and environmental adaptation.

### 2.6. Prediction of Secondary Metabolite Gene Clusters

AntiSMASH v7.1.0 [30] online platform was employed to predict and describe biosynthetic gene clusters (BGCs) responsible for secondary metabolite production. The analysis used default parameters with Detection strictness set to “relaxed” and Prodigal for gene annotation. The Known Cluster Blasts module identified BGCs homologous to known clusters in the MIBiG database, facilitating detection of antimicrobial and biocontrol-related compounds.

BiG-SCAPE v1.1.5 [31] was used with glocal alignment mode, GCF threshold of 0.3, and the MIBiG database to categorize BGCs into gene cluster families (GCFs). The analysis was performed in a conda environment (big-scape) with Python 3.11 and hmmer installed via bioconda. The Pfam database (v35.0) was downloaded and pre-processed using hmmpress. For detailed installation and analysis commands, see Supplementary Methods S1.

RStudio v2024.12.1 and R v4.4.3 were utilized for data analysis and clustered heatmap generation. Visualizations were generated using the ggplot2 package v3.5.2 with custom parameters for bubble plots, including size scaling by similarity and color coding by cluster number. For the complete R script, see Supplementary Methods S1. This workflow established a foundation for linking genetic variation to the biocontrol efficacy of *B. brevis*.

## 3. Results

### 3.1. Genomic Characteristics of Brevibacillus brevis

The genomic analysis of 25 strains of *B. brevis* reveals remarkable genomic diversity, reflecting the species’ adaptability within its ecological niche. All genomes met predefined quality thresholds (completeness ≥90%, contamination ≤5%), with none excluded for failing these criteria. Among these, nine genomes have been assembled to a complete level, while the rest are at the scaffold or contig level, ensuring robust data for comparative analysis. Genome sizes range from 5.92 Mb (strain 3) to 6.73 Mb (strains CCM 2050 and NCTC2611). Except for strain NEB573, the GC content of most strains varies narrowly between 47.0% and 47.5%. Based on uniform annotation with Prokka v1.14.6, the number of coding sequences (CDS) ranges from 5463 (strain LABIM17) to 6334 (strain CCM 2050), indicating differences in gene content (Table 1). These genomic features align with the ecological versatility of *B. brevis*. Larger genomes and higher CDS counts potentially encode diverse metabolic and biological control functions. The strains originate from various environments, including rhizospheric soils (e.g., tobacco, wheat, watermelon, and tea plant), bulk soils, and unique niches such as air filters and rainforest soils, reflecting the species’ ecological adaptability.

**Table 1:** Genomic features of the 25 *Brevibacillus brevis* strains analyzed in this study.

Strain	Size (Mb)	GC (%)	Assembly Level	CDS	Source	Country	Accession Number	Reference
3	5.92	47.5	Scaffold	5735	-	Germany	GCA_903797695.1	-
Ag35	6.48	47.5	Contig	5905	Alfalfa root nodule	USA	GCA_014526365.1	[32]
ATCC 35690	6.13	47.5	Contig	5563	Soil	Poland	GCA_002161835.1	-
B011	6.16	47.5	Complete	5582	Tobacco rhizosphere	China	GCA_022026395.1	[33]
CCM 2050^T^	6.73	47.5	Contig	6334	-	China	GCA_042682345.1	-
DSM 30^T^	6.61	47.5	Scaffold	6273	-	USA	GCA_003385915.1	-
DZQ7	6.44	47.5	Complete	5864	Tobacco rhizosphere soil	China	GCA_001039275.2	[34]
FJAT-0809-GLX	6.02	47.5	Contig	5541	Watermelon rhizosphere soil	China	GCA_000346255.1	[17]
G25-137	6.34	47.0	Scaffold	5894	-	China	GCA_015912885.1	-
GZDF3.1	6.43	47.0	Scaffold	5951	Soil	China	GCA_001649505.1	-
HK544	6.49	47.5	Complete	5918	Soil	South Korea	GCA_007725005.1	[35]
HNCS-1	6.35	47.0	Complete	5776	Tea rhizosphere soil	China	GCA_030377165.1	[36]
I2-B3	6.22	47.5	Contig	5779	Air filter	USA	GCA_019749035.1	-
LABIM17	5.95	47.5	Chromosome	5463	Rainforest soil	Brazil	GCA_021401445.1	[37]
MGMM11	6.32	47.0	Complete	5776	Wheat rhizosphere soil	Russia	GCA_029958365.1	-
NBRC 100599	6.30	47.5	Complete	5807	-	Japan	GCA_000010165.1	-
NBRC 110488	6.28	47.5	Scaffold	5868	-	Japan	GCA_001748185.1	-
NBRC 15304^T^	6.52	47.5	Contig	6193	-	Japan	GCA_006539845.1	[38]
NCTC2611^T^	6.73	47.5	Complete	6286	-	-	GCA_900637055.1	-
NEB573	6.23	54.0	Complete	5868	-	New England	GCA_031583145.1	-
NPDC077532	6.37	47.0	Scaffold	5948	-	USA	GCA_044542915.1	-
NRRL NRS-604^T^	6.61	47.5	Contig	6255	Soil	USA	GCA_003012835.1	-
SDF0063	6.24	47.5	Contig	5716	Soil	Brazil	GCA_006864225.1	[39]
X23	6.64	47.0	Complete	6213	Vegetable soil	China	GCA_000296715.2	[16]
YSY-3.4	6.19	47.5	Contig	5657	Dipterocarp forest soil	Vietnam	GCA_045866595.1	[40]

### 3.2. Phylogenetic Structure and Evolutionary Relationships Within Brevibacillus brevis

**Figure 1:**
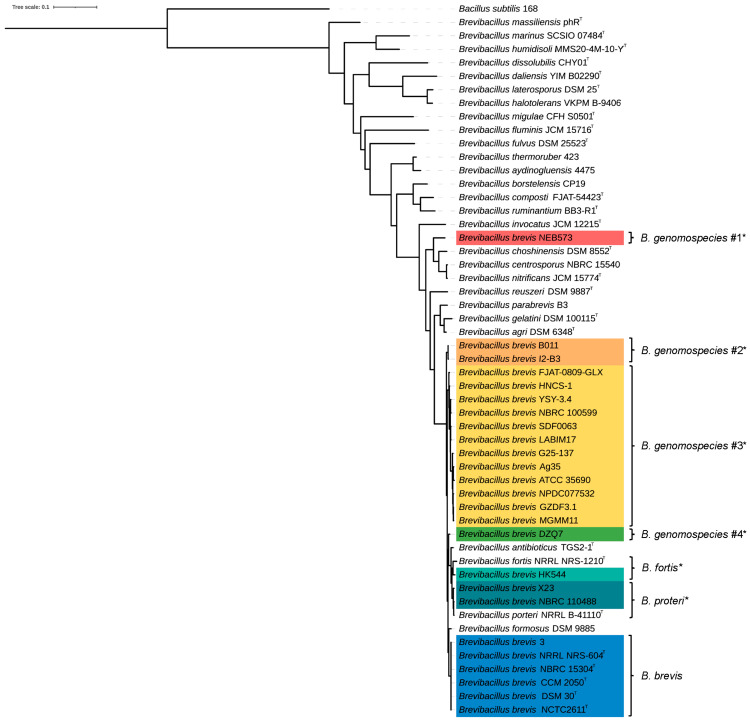
Maximum likelihood phylogenetic tree of *Brevibacillus* strains based on whole-genome sequences. The tree was constructed using 25 *B. brevis* genomes, 27 genomes from other *Brevibacillus* species, and an outgroup (*Bacillus subtilis* 168). The inclusion of *B. subtilis* 168 as an outgroup provided a stable root for the phylogeny, ensuring accurate resolution of lineage-level relationships. * indicates that the *B. brevis* strain(s) in this clade are not clustered with the type strain.

A phylogenetic tree was reconstructed using the whole-genome sequences of 25 *B. brevis* strains, 27 representative genomes from other *Brevibacillus* species, and *Bacillus subtilis* 168 as an outgroup (Figure 1). The topology resolved the 25 *B. brevis* strains into seven well-supported lineages: *B. genomospecies* 1 (NEB573), *B. genomospecies* 2 (B011 and I2-B3), *B. genomospecies* 3 (Ag35, ATCC 35690, FJAT-0809-GLX, etc.), *B. genomospecies* 4 (DZQ7), *B. fortis* (HK544), *B. porteri* (X23 and NBRC 110488), and *B. brevis* (including strains 3, CCM 2050, DSM30, etc.). Within this structure, *B. genomospecies* 1 (NEB573) formed a distinct lineage, while the remaining 24 strains grouped into a major clade comprising the other six lineages, reflecting evolutionary diversification. To rigorously test the hypothesis that NEB573 represents a novel *Brevibacillus* species, we performed Average Nucleotide Identity (ANI) against 28 other *Brevibacillus* strains (Supplementary Table S2). NEB573 exhibited ANI values below 82% when compared with all tested species, confirming its divergence from known *Brevibacillus* lineages. Notably, its closest relative was *B. choshinensis* DSM 8552ᵀ (ANI = 81.25%), further supporting its status as a novel species.

### 3.3. Genomic Divergence Inferred from ANI and dDDH Metrics

**Figure 2:**
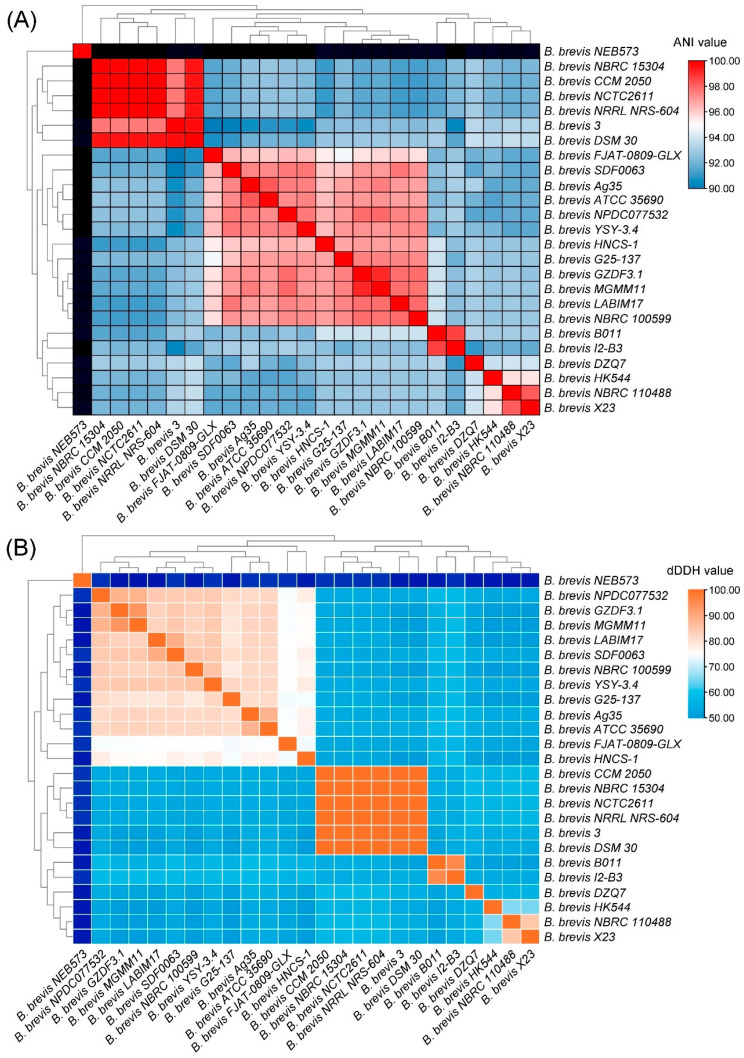
ANI and dDDH analyses of the 25 *Brevibacillus brevis* strains. (**A**) The heatmap illustrates the ANI values among the 25 *B. brevis* strains, where gray represents missing values; (**B**) the heatmap displays the dDDH values among the 25 *B. brevis* strains.

Building on the phylogenetic relationships, we next assessed genetic diversity using ANI and dDDH analyses to refine taxonomic boundaries. ANI and dDDH analyses were conducted to delineate the species boundaries and assess intra-species diversity among the 25 *B. brevis* strains. ANI values above 95% and dDDH values exceeding 70% are typically used as thresholds for species-level classification [41]. Based on these criteria, the strains were grouped into six distinct populations, reflecting notable genomic divergence (Figure 2). Several strains-including NBRC 15304, CCM 2050, NCTC2611, NRRL NRS-604, DSM 30, and strain 3-exhibited high similarity, forming a coherent subgroup.

Notably, strains HK544 (classified as *B. fortis*) and X23/NBRC 110488 (classified as *B. porteri*) were resolved as distinct lineages in the phylogenetic tree (Figure 1). Their ANI values (95.56% and 95.64%) fall within the 95% “species boundary gray zone”, where genomic similarity alone is insufficient for definitive taxonomic assignment. This gray zone necessitates complementary evidence to resolve species-level relationships. Here, the dDDH values (64.9% and 65.6%) provide crucial supporting evidence, as both values are below the 70% threshold, reinforcing their classification as distinct species.

In contrast, strain NEB573 demonstrated pronounced divergence, with ANI and dDDH values falling below the accepted thresholds. These findings strongly suggest that NEB573 may constitute a novel species within the *Brevibacillus* genus. This conclusion aligns with prior work by Du et al. (2024) [12], who similarly proposed NEB573 as a potential new species based on genomic analyses. Our results further support this hypothesis through comprehensive phylogenetic and genomic divergence assessments across a larger strain cohort (n = 25).

### 3.4. Pan-Genome Structure and Dynamics

**Figure 3:**
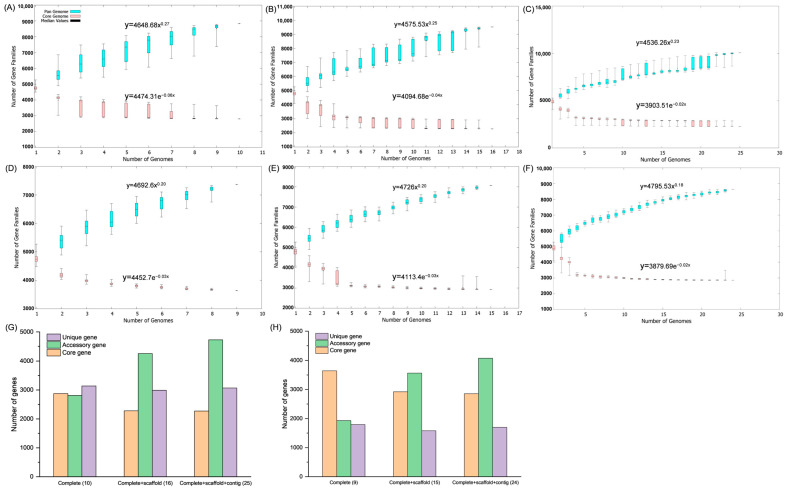
Pan-genome analysis of *Brevibacillus brevis*. The curves in panels (**A**–**F**) are generated based on Heap’s Law and the least-squares fit of exponential regression decay. (**A**–**C**) present graphs showing the core and pangenome sizes for the complete genomes, complete + scaffold, and complete + scaffold + contig genomes of 25 strains, respectively. (**D**–**F**) exclude strain NEB573 and display stable core and pan-genome trends. (**G**,**H**) illustrate the number of core gene clusters, accessory gene clusters, and unique gene clusters for 25 strains and for all strains except NEB573, respectively. The number in brackets of the x-axis scale label represents the number of genomes analysed.

Pan-genome analysis was performed using all 25 genomes, with comparisons across subsets defined by assembly level (complete, scaffold, contig) and by excluding strain NEB573. Assembly level had minimal impact on pan-genome trends (Figure 3A–F). As anticipated, increasing genome numbers resulted in minor fluctuations in core and unique gene counts but a substantial increase in accessory genes (Figure 3G). Exclusion of NEB573 significantly elevated the core gene count while reducing unique genes (Figure 3D–F,H), indicating its substantial contribution to strain-specific genes.

Consequently, downstream analyses utilized the 24-strain dataset (excluding NEB573). This refined dataset comprised 8631 total gene clusters: 2855 (33.08%) core genes, 4077 (47.24%) accessory genes, and 1699 (19.68%) unique genes. Heap’s Law modeling (y = 4795.53x^0.18^)—fitted via least-squares regression of exponential decay—demonstrated that the number of gene families increased incrementally with each added genome. The calculated b = 0.18 (α = 1 − b = 0.82 < 1) confirms an open pan-genome.

### 3.5. Functional Insights from Gene Family Annotations

**Figure 4:**
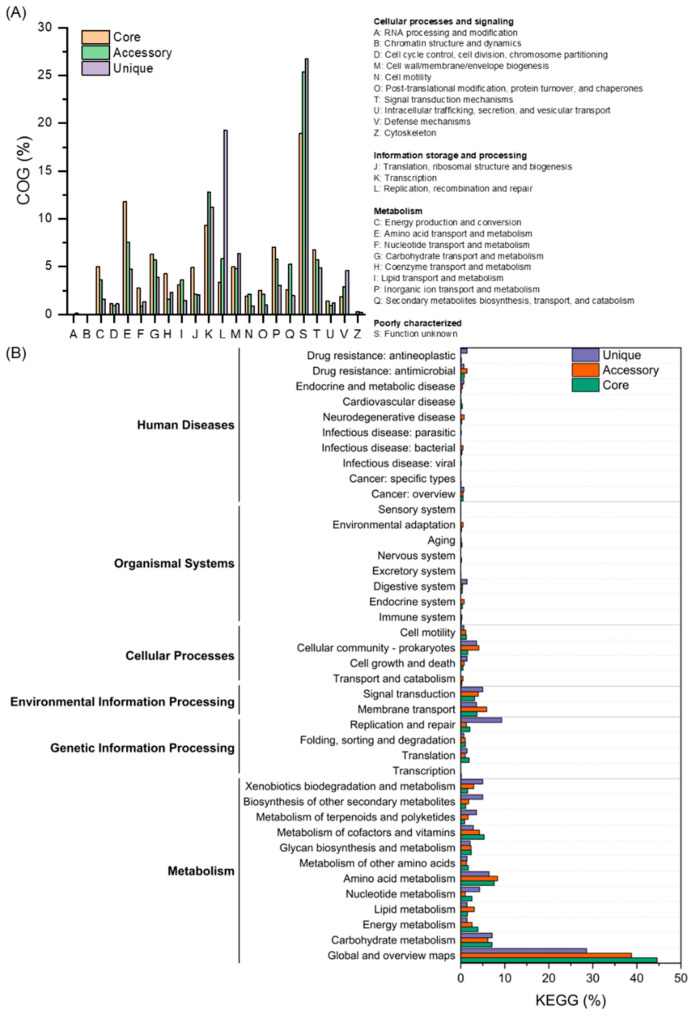
Functional annotation of *Brevibacillus brevis* gene families. (**A**) Distribution of Clusters of Orthologous Groups (COG) categories for core, accessory, and unique genes. (**B**) KEGG pathway analysis highlighting metabolic contributions of gene families.

Functional annotations were performed based on the pangenome analysis results of 24 *B. brevis* strains, excluding strain NEB573. The annotations of gene families provided offered new perspectives on the metabolic and ecological roles of *B. brevis*. In the whole-genome analysis using Clusters of Orthologous Groups (COG), significant differences were observed in the COG categories among the core, accessory, and unique gene families. Genes of unknown function comprised the largest category for core, accessory, and unique genes, accounting for 18.92%, 25.34%, and 26.71%, respectively (Figure 4A). Additionally, in terms of COG categories, most genes in the core genome were essential for life activities, such as amino acid transport and metabolism (E) (11.81%), transcription (K) (9.34%), and inorganic ion transport and metabolism (P) (7.03%). For the accessory genome, COG annotations showed that the largest categories were transcription (K) (12.81%), amino acid transport and metabolism (E) (7.56%), and replication, recombination, and repair (L) (5.83%), indicating its role in adapting to environmental signals. The unique gene families were enriched in replication, recombination, and repair (L) (19.27%), transcription (K) (11.22%), and cell wall/membrane/envelope biogenesis (M) (6.34%), suggesting specific adaptations of the strains to environmental pressures and microbial competition.

The functions of core, accessory, and unique genes were primarily focused on metabolism, accounting for 80.28%, 74.20%, and 69.29%, respectively. The proportion of genetic information processing in unique genes (11.43%) was significantly higher than that in the core genome (5.15%) and accessory genome (3.34%), with a focus on replication and repair (9.29%) (Figure 4B).

### 3.6. Diversity of Secondary Metabolite Biosynthetic Gene Clusters

A predictive analysis revealed that all the strains of *B. brevis* harbour a wealth of secondary metabolite biosynthetic gene clusters in their genomes. The number of gene clusters did not correlate well with the genome size, and the potential bioactive substances encoded by these clusters exhibited diversity. Among the 24 *B. brevis* genomes, 462 BGCs were predicted, including 153 non-ribosomal peptide synthetases (NPRSs) and 79 hybrid polyketide synthase-nonribosomal peptide synthetase enzymes (PKS-NRP hybrids). Furthermore, strains 3, CCM2050, NPDC077532, and YSY-3.4 have the highest number of predicted BGCs, with each strain harbouring more than 30; the remaining strains have fewer than 20 predicted gene clusters.

**Figure 5:**
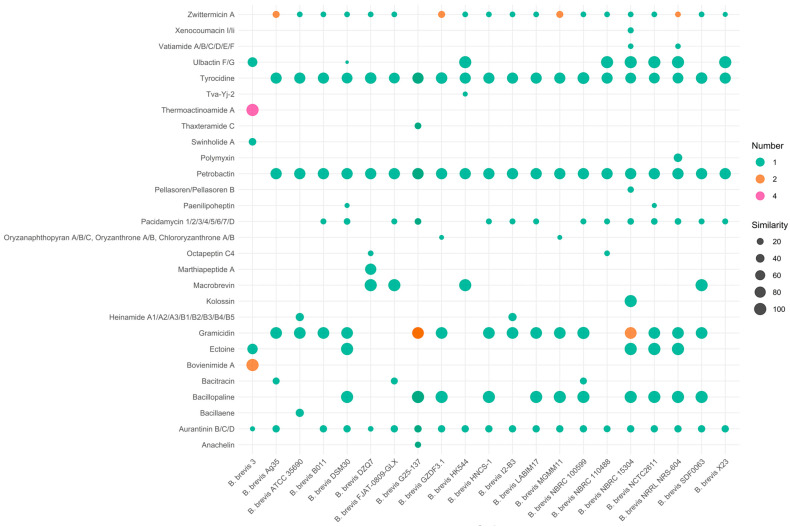
Heatmap of known compound-related BGCs in 21 *Brevibacillus brevis* strains. Heatmap illustrating the distribution and similarity of the 28 antiSMASH-predicted BGCs encoding known antimicrobial compounds across strains, with intensity indicating presence and similarity to reference clusters.

With the exception of three strains (strain CCM 2050, NPDC077532, and YSY-3.4), the gene clusters in the genomes of the other 21 strains shared homology with BGCs associated with 28 known compounds (Figure 5). Most genomes contained gene clusters similar to those involved in the synthesis of compounds such as zwittermicin A, petrobactin, pacidamycin 1/2/3/4/5/6/7/D, tyrocidine, gramicidin, bacillopaline, and aurantinin B/C/D, indicating potential for combinatorial biosynthesis. Among these, the biosynthetic gene clusters for tyrocidine, petrobactin, gramicidin, and bacillopaline exhibited a high degree of similarity, with a similarity rate exceeding 75%. Although compounds such as ulbactin F/G, macrobrevin, kolossin, bacillopline, and ectoine are present only in specific strains, they share 100% similarity. This diversity of BGCs reveals an underexplored chemical arsenal with significant potential for biotechnological exploitation.

## 4. Discussion

*B. brevis* has attracted increasing attention as a promising biocontrol bacterium due to its broad-spectrum antimicrobial activities, ecological versatility, and potential applications in sustainable agriculture [42]. The present study expands this understanding by systematically characterizing the genomic features, pangenome structure, and secondary metabolic potential of 25 *B. brevis* strains. These findings highlight not only the species’ genomic diversity but also its implications for biocontrol applications, thereby providing new perspectives for developing effective microbial agents against plant.

The comparative genomic analysis revealed an average genome size of 6.35 Mb in *B. brevis*, larger than that of most other species in the genus, suggesting greater metabolic versatility and ecological adaptability [43]. Except for strain NEB573, the GC content among strains was highly conserved (47.0–47.5%), indicating close evolutionary relationships. Whole-genome phylogenetic reconstruction, supported by ANI and dDDH analyses, clustered the strains into seven distinct clades, consistent with high-resolution genomic taxonomy [44,45,46]. Notably, strain NEB573 displayed low similarity indices and independent phylogenetic placement, supporting its candidacy as a novel species. This observation underscores the genetic heterogeneity within *B. brevis* and highlights its ongoing evolutionary diversification.

Pangenome analysis further confirmed that the *B. brevis* pan-genome is open, reflecting its capacity for horizontal gene transfer [47]. Such genomic plasticity likely underpins the species’ ability to colonize diverse ecological niches, including rhizosphere soil, plant surfaces, marine habitats, and artificial environments. The core genome, comprising 2855 gene families (33.08% of the pan-genome), encodes essential functions such as amino acid transport and metabolism, ensuring metabolic stability across strains. In contrast, the 1699 unique genes (19.68%) contribute to niche-specific adaptations, including DNA repair pathways associated with stress tolerance and microbial competition [48]. The removal of strain NEB573 from the dataset markedly increased the number of core gene families, further reinforcing its distinctiveness from the rest of the species. These results collectively highlight the ecological flexibility and adaptive strategies that enable *B. brevis* to thrive under diverse and dynamic conditions.

A particularly notable finding was the exceptional diversity of secondary metabolite biosynthetic gene clusters (BGCs) across *B. brevis* strains. antiSMASH analysis revealed multiple clusters encoding NRPSs, PKSs, siderophores, and bacteriocins. This chemical repertoire, enriched in bacteriocins and siderophores, underscores the species’ unique adaptability to diverse ecological niches and its potential as a biocontrol agent. Compounds such as tyrocidine and gramicidin are well-known for disrupting microbial membranes through pore formation, leading to cytoplasmic leakage and cell death, with demonstrated efficacy against pathogens like Staphylococcus aureus and partial activity against Candida albicans [49]. Likewise, siderophores such as petrobactin and bacillopaline sequester Fe^3+^ with high affinity, enabling *B. brevis* to outcompete pathogens under iron-limited conditions typical of the rhizosphere [50]. Interestingly, edeine, a translation-inhibiting antibiotic previously reported in *B. brevis*, was not detected in the present dataset, suggesting limitations of genome mining tools and highlighting the need for experimental validation [10,36]. This highlights the importance of integrating genomic predictions with metabolomic profiling to comprehensively uncover secondary metabolic repertoires. Importantly, the distribution and diversity of BGCs among *B. brevis* strains appear largely independent of phylogenetic relationships; strains within the same clade do not necessarily share identical BGC profiles, and some distantly related strains exhibit highly similar clusters, suggesting that horizontal gene transfer and niche-specific selective pressures are key drivers of secondary metabolite diversification.

By situating these findings within the broader literature, this study emphasizes both the novelty and applied significance of *B. brevis* genomics. Compared with related *Bacillus* species, which typically harbor 5-15 BGCs, *B. brevis* exhibits an average of 19.25 BGCs per strain—nearly 28% higher than the upper limit of this range [51]. This genomic richness, combined with evidence of ecological adaptability, positions *B. brevis* as a valuable candidate for agricultural and medical biocontrol strategies. Integrating strains enriched in distinct BGCs into multi-strain consortia could enhance efficacy against complex soil-borne diseases such as *Fusarium* wilt and *Rhizoctonia* rot [52]. At the same time, challenges remain, including strain stability in field formulations, ecological safety of environmental release, and the regulatory hurdles of biocontrol commercialization. Future directions should therefore focus on multi-omics approaches to validate BGC expression under relevant ecological conditions, coupled with field trials to assess real-world performance. By bridging genomic insights with practical applications, this work establishes *B. brevis* as a versatile and innovative candidate for sustainable biocontrol solutions.

## Data Availability

The data presented in the study are openly available in the NCBI GenBank Database (https://www.ncbi.nlm.nih.gov/genome, accessed on 20 March 2025). Publicly available datasets were analyzed in this study. The accession numbers of the genomes used are listed in Table 1 and Supplementary Table S1.

## Supplementary Materials

The following supporting information can be downloaded at: https://www.mdpi.com/article/10.3390/microorganisms13112456/s1, Table S1. Genomic characteristics of reference genomes from *Brevibacillus* species used in the phylogenomic analysis. Table S2. Pairwise ANI values among 29 strains of the *Brevibacillus* genus (%). Supplementary Methods S1. Analysis commands. Supplementary File S1. Maximum likelihood phylogenetic tree of *Brevibacillus* strains based on whole-genome sequences.
